# The Programme of the Hospital Saturday Fund

**Published:** 1891-07-11

**Authors:** 


### PASSING TOPICS.

### THE PROGRAMME OF THE HOSPITAL SATURDAY FUND.

The observations made in The Hospital for the current week by Mr. Reginald B. D. Acland seem to call for a passing notice, and we may say at once that if the article which appeared under this heading upon the 27th ult., mis-stated in any particular what has fallen from his lips, Mr. Acland is fully entitled to an apology.

We do not, however, gather that the facts and the comments offered lie far apart, and though, of course, we are bound to believe with Mr. Acland's statement before us that some slender verbal inaccuracy has contrived to creep in, a diligent search has not enabled us to detect anything of gravity enough to affect the main issues which, as easily as not, might have been dealt with irrespective altogether of the evidence referred to in the article.

The Chairman of the Saturday Fund tells your readers that "'Agricola' suggests I gave some evidence to the effect that the authorities of the Fund are bent upon exacting a *quid pro quo* in return for any assistance they render," and he adds that he never said or suggested anything of the kind. Well, we have not said he did, nor is it likely he would, but that it is a cardinal point with the Saturday Fund authorities to get all they can is a matter of common knowledge. It was upon that we commented, and if Mr. Acland does not see that by admitting "we receive letters of recommendation in proportion to our grants, sometimes on the same scale as that on which they are given to ordinary subscribers and sometimes according to a special scale," he surrenders the whole position, it is only because he fails to appreciate what the demand of his Society amounts to.

The system of granting letters of recommendation is at best logically indefensible. Hospitals in return for gifts accept obligations altogether disproportionate and which the managers are perfectly conscious they would be unable to meet. The system is no less morally than commercially unsound, but it is supposed to encourage subscriptions, and is made possible by the fact that scarcely any ordinary donor makes full use of his privileges. In the case of the Saturday Fund, there is a probability, equivalent to a certainty, that every letter will be apportioned and made to do duty; that is to say, the Fund will require in return for every guinea of its grant several guineas' worth of relief for its nominees.

With this notorious fact in full view of all whose eyes are open, who can question the propriety of a criticism directed against the theory that contributors to the Saturday Fund acquire a right to interfere in the management of the Institutions? Mr. Acland says he disclaimed the desire to "meddle," and once again we must appeal from phraseology to fact. The Saturday Council give plain notice that their grant to any individual hospital will be influenced by the replies to certain specified questions included in the form issued to institutions seeking to participate. As these enquiries have to do with the acceptance of working-class nominees as governors and members of committee, the evening attendances of physicians, and, we believe, the examination of any or all of the hospital books, it seems more reasonable and dialectically correct for the hospitals to call this kind of thing "meddling" than it is for Mr. Acland to say his Society only desires to "assist."

Mr. Acland claims for the Saturday Fund that it has distributed its "money in such a way that those institutions where the largest amount of work is the most economically and efficiently performed receive the largest grants." We do not for a moment doubt that the Council have tried to do this, but we are equally certain that the verdict of competent judges is that they have failed. The question is a large one, and has been previously dealt with in this column. Suffice it to say now that the method of distributing the Fund, however much it may have satisfied the managers of some petty and questionable hospitals which have received grants out of all proportion to the extent or value of their work, has not commended itself to general approval. Agricola.

